# Swedish dentists’ use of pharmacological pain management in children: a survey

**DOI:** 10.1007/s40368-025-01082-x

**Published:** 2025-07-16

**Authors:** R. Roxner, H. Berlin, G. Klingberg

**Affiliations:** Department of Pediatric Dentistry, Faculty of Odontology, Malmö University, SE-205 06 Malmö, Sweden

**Keywords:** Pain, Pain management, Local anesthesia, Child, Dentist, Pediatric dentistry

## Abstract

**Purpose:**

The aim of this cross-sectional study was to explore how Swedish General Dental Practitioners (GDPs) and Specialists in Paediatric Dentistry (SPDs) use pharmacological pain management, focusing on local anesthetics (LA) when treating children.

**Methods:**

582 GDPs in southern Sweden and 137 SPDs nationwide received a questionnaire with 4 clinical scenarios covering filling therapy and tooth extractions in children. Each scenario had questions about how often the dentist would use LA and topical anesthetics, answered on a 5-point Likert-type scale (*Always*, *Often*, *Sometimes*, *Seldom*, *Never*).

**Results:**

The overall response rate was 48.0% (243 GDPs and 102 SPDs). Use of LA reported as *Always* or *Often* was more common in SPDs than GDPs for filling therapy in primary molars (98.0% vs. 90.9%, *p* = 0.019) as well as in permanent molars (99.0% vs. 91.7%, *p* = 0.006). GDPs who reported *Always* or *Often* using LA for filling therapy in primary teeth were younger (42.2 years vs. 49.1 years, *p* = 0.004) and had fewer years of experience as a dentist (14.2 years vs. 19.9 years, *p* = 0.016) compared with GDPs reporting less frequent use.

**Conclusion:**

There was an underuse of LA among GDPs when treating children. The reasons for refraining from LA are not fully understood, but possible contributing factors can be identified within work environment, insufficient undergraduate training and lack of organizational support and guidelines.

## Introduction

Children frequently perceive dental treatment as being painful (Nakai et al. 2000; Krekmanova et al. 2009; Ghanei et al. 2018; Boyd et al. 2023), which is problematic as painful dental treatment is a risk factor for developing dental fear and anxiety (Skaret et al. 1998, 1999; Raadal et al. 2002; Klingberg and Broberg 2007; Tickle et al. 2009). In a longer perspective, dental anxiety can lead to avoidance of dental care (Skaret et al. 1999; Berge et al. 2016), deterioration of dental health (Skaret et al. 2000; Wigren et al. 2009), and have a negative impact on oral health-related quality of life in children (Alharbi et al. 2021). At the same time, several studies have reported an underuse of local anesthetics (LA) when dentists treat children (Klingberg et al. 1994; Milgrom et al. 1994; Murtomaa et al. 1996; Wondimu and Dahllöf 2005; Rønneberg et al. 2015). When discussing the underuse and how to increase the use of LA, information and educational activities are often mentioned. A need for more knowledge about pain and pain management in children and adolescents among both dentists (Berlin et al. 2018; Krekmanova et al. 2021) and dental students (Jaldin and Krekmanova 2023; Roxner et al. 2024) is suggested in studies from Sweden. Based on these findings, pain issues have been more focused on in both undergraduate training and continuous educational activities. As the experience of procedural pain in child dental care is a potential health hazard, it is important to repeat surveys on the use of LA. This serves two purposes, to draw attention to the problem and to evaluate and develop strategies to improve pain prevention in dental care. Therefore, the aim of the present study was to explore how Swedish General Dental Practitioners (GDPs) and Specialists in Paediatric Dentistry (SPDs) use pharmacological pain management, with special focus on LA, when treating children. Based on the aim, the null hypotheses were that there are no differences in use of LA (i) between GDPs and SPDs, and (ii) among GDPs when treating primary or permanent teeth.

This report was written in accordance with the STROBE statement (Vandenbroucke et al. 2007).

## Methods

### Questionnaire

A questionnaire about demographics, dental education, occupational situation, and four potentially painful clinical scenarios was used. The scenarios focused on pharmacological pain management strategies and were adapted from Wondimu and Dahllöf (2005) and have previously been used by Berlin et al. (2018). The scenarios were: (1) filling therapy in tooth 55 in a 5-year-old, (2) filling therapy in tooth 16 in a 10-year-old, (3) extraction of tooth 51 in a 4-year-old, and (4) extraction of tooth 14 in a 12-year-old. For each scenario, dentists were asked about frequency of using a topical anesthetic, LA, and conscious sedation. Answers were given on a five-point Likert-type scale: *Always*, *Often*, *Sometimes*, *Seldom*, or *Never*. For the statistical analyses, Likert-scale responses were dichotomized into *Always–Often* and *Sometimes–Seldom–Never*, respectively. Similarly, the variable *years of experience as a dentist* was dichotomized as ≤ 5 years or > 5 years and the variable *hours treating child patients per week* as ≤ 10 h or > 10 h per week.

Information about the study, a written consent form, and the questionnaire were sent out by post together with a pre-paid return envelope. To facilitate the response, the dentists could choose to either answer the questionnaire by mail or by filling out a digital version. Dentists not providing dental care to children were asked to mark this in the questionnaire and return it, by mail or digitally. Two reminders were sent after four and twelve weeks, respectively. The first mailing was sent in April 2023, and the last questionnaire was returned by September 2023.

### Ethics

Ethical approval for the present study was obtained from the Swedish Ethical Review Authority [Göteborg 2021-04961]. The study protocol was registered in ClinicalTrials.gov (NCT05911542).

### Setting

In Sweden, comprehensive dental care (including specialist dental care), free-of-charge, was at the time of the survey offered to children, adolescents, and young adults up to and including the calendrical year individuals turn 23 years. Dental care for children is carried out by either Public Dental Service (PDS) or Private Practitioners (PPs). The PDS is responsible for the majority of children within general dental care at national level approximately 86% (SOU 2021). SPDs are mainly organized under PDS and offer dental care after referrals. In general, dentists work 40 h per week, which is the length of a working week for full-time employment. Reimbursement for child dental care provided by GDPs is based on fixed capitated payments, whereas a fee-for-service model is applied for the majority of dental care for healthy adult patients.

Sweden is divided into 21 counties and has approximately 10.5 million inhabitants, of which 2.4 million are 0–19 years old, according to official statistics (Statistics Sweden). The county of Scania, where data from GDPs were collected, is in the far south of Sweden and the third largest county by population. Scania is home to 1.4 million people, of which approximately 330 thousand are between the ages of 0 and 19 years, representing just over 13% of the national population (Statistics Sweden). Malmö, the largest city in the county and third in Sweden, is the county seat and has a university with one of the four Swedish dental schools. In 2021, the total number of dentists nationwide was 8,066, with 1,078 located in Scania, representing slightly more than 13%. There are nine recognized dental specialties in Sweden, and just over 12% of dentists are licensed specialists (Socialstyrelsen 2023a). Of the 137 clinically active SPDs (including post-graduate dentists), 17 were active in Scania at the time of data collection. Dental health among children and adolescents in Scania does not differ substantially from the national average (Socialstyrelsen 2024).

### Participants

GDPs in the county of Scania and Swedish SPDs nationwide were identified through the Register of Authorised Healthcare Professionals (HOSP), held by the National Board of Health and Welfare. Dentists undergoing specialist training in pediatric dentistry were included in the SPD group. Inclusion criteria for GDPs were ≤ 65 years of age and working in a clinic providing child dental care. Information was limited to clinic level and there was no information whether an individual dentist actually accepted children for dental care or not. Thus, GDPs working in clinics accepting child patients were assumed to be treating children and eligible for this survey study. Inclusion criteria for SPDs were ≤ 65 years of age and treating child patients in the clinic. Exclusion criteria for both GDPs and SPDs were > 65 years of age or not working with child dental care in clinical practice.

### Statistical analyses

Data were analyzed using IBM SPSS Statistics for Windows (version 28.0). Dichotomous variables were analyzed by chi-square test for independence. When needed, Fisher’s exact test was applied. To analyze individual dentists’ reported use of pain management in younger compared to older children (e.g., LA in conjunction with filling therapy in a 5-year-old compared to a 10-year-old), McNemar’s Test was used. Student’s t test was used to analyze differences in age and years of experience as a dentist. To compare differences between participants and non-participants, Student’s t test and Mann–Whitney U test were employed. Direct binary logistic regressions were performed to evaluate the impact of a set of predictor variables on GDPs’ reported use of LA when performing filling therapy. Independent variables in the model were: *sex*, *years of experience as a dentist*, *workplace*, *dentists having children of their own*, and *number of hours treating children per week*. Significance level was set at *p* < 0.05.

## Results

A total of 1096 GDPs and 153 SPDs were identified through the HOSP registry. In total, 514 GDPs reported not treating children or worked in clinics not accepting child patients and were excluded. For 98 GDPs, it was not possible to determine whether they worked in clinics providing dental care to children, and to avoid inaccurate exclusion, it was decided they should be included. Finally, 582 GDPs were identified as working in clinics providing dental care to children and eligible. Importantly, it could not be determined whether all of them actually treated children themselves. For SPDs, 16 were reported as not active as clinicians. Thus, finally 137 SPDs were eligible.

In all, 48.0% (345 dentists; 243 GDPs, 102 SPDs) participated. Characteristics of non-responding, and responding dentists are shown in Table 1. The response rate was higher for SPDs than for GDPs (74.5% vs. 41.8%, *p* < 0.001). Age did not differ between non-responders and responders (neither SPDs nor GDPs). Regarding sex, there were no differences between responding and non-responding SPDs, while more female than male GDPs participated (46.4% vs. 32.5%, *p* = 0.002). Of GDPs, 42.3% worked in PDS and 57.7% as PPs. PDS-employed GDPs answering the survey were on average younger than those working as PPs (40.5 years vs. 44.7 years, *p* = 0.003). GDPs who reported > 5 years of experience as a dentist reported working as PPs more frequently than in PDS (63.8% vs. 36.2%, *p* = 0.001). Working > 10 h per week with child patients was more common in GDPs with ≤ 5 years of experience as a dentist compared to GDPs with > 5 years of experience (53.7% vs. 34.4%, *p* = 0.001).

**Table 1:** Characteristics of responding and non-responding dentists

	GDPs				SPDs			
	Total	Female	Male	*p* value	Total	Female	Male	*p* value
*Respondents*								
*n* (%)	243 (100)	180 (74.1)	63 (25.9)		102 (100)	87 (85.3)	15 (14.7)	
Mean age (SD)	42.8 (10.8)	41.9 (10.6)	45.7 (11.2)	0.016^a^	46.3 (9.8)	46.5 (9.5)	45.4 (11.5)	0.688^a^
≤ 5 yrs experience as a dentist, *n* (%)	54 (22.3)	42 (17.3)	12 (5.0)	0.584^b^				
> 10 h treating children per week, *n* (%)	94 (39.0)	81 (33.6)	13 (5.4)	0.001^b^				
Work in PDS, *n* (%)	101 (41.6)	84 (34.6)	17 (7.0)	0.009^b^				
*Non-respondents*								
*n* (%)	339 (100)	208 (61.4)	131 (38.6)		35 (100)	29 (82.9)	6 (17.1)	
Mean age (SD)	42.3 (11.6)	42.1 (11.6)	42.7 (11.5)	0.650^a^	44.3 (8.9)	43.0 (7.83)	50.7 (11.47)	0.051^a^

GDPs = General Dental Practitioners, SPDs = Specialists in Paediatric Dentistry, PDS = Public Dental Service

^a^Student’s t test

^b^Chi-square test

Table 2 shows the dentists’ responses to the four clinical scenarios. Overall, SPDs reported more use of pharmacological strategies than GDPs except for LA in conjunction with extractions, which was equally used in both groups (Table 3). McNemar’s tests showed no differences in GDPs’ use of LA between treating primary and permanent teeth (filling therapy *p* = 0.824, extraction *p* = 1.000). GDPs who reported use of LA *Always* or *Often* for filling therapy in primary teeth were younger (42.2 years vs. 49.1 years, *p* = 0.004) and had fewer years of experience as a dentist (14.2 years vs. 19.9 years, *p* = 0.016) compared with GDPs reporting use of LA *Sometimes*, *Seldom* or *Never*. There were no such differences regarding filling therapy in permanent teeth. No statistically significant differences between GDPs employed in PDS or working as PPs were observed regarding reported use of LA. Female and male GDPs reported equal use of LA in conjunction with filling therapy in tooth 55, whereas in a permanent molar, female GDPs reported giving LA more frequently than males (93.8% vs. 85.5%, *p* = 0.041). For topical anesthetic, dentists reported an overall high use, whereas conscious sedation was used more frequently by SPDs.

**Table 2:** GDPs’ (General Dental Practitioners) and SPDs’ (Specialists in Paediatric Dentistry) reported use of pharmacological pain management and conscious sedation in four clinical scenarios

	GDPs					SPDs				
	Always	Often	Sometimes	Seldom	Never	Always	Often	Sometimes	Seldom	Never
	*n* (%)	*n* (%)	*n* (%)	*n* (%)	*n* (%)	*n* (%)	*n* (%)	*n* (%)	*n* (%)	*n* (%)
*Scenario*										
Filling therapy tooth 55 in a 5 y.o										
How often do you use…										
Topical anesthetic	207 (85.2)	26 (10.7)	6 (2.5)	3 (1.2)	1 (0.4)	99 (97.1)	3 (2.9)	0	0	0
Local anesthetic	160 (66.1)	60 (24.8)	19 (7.9)	3 (1.2)	0	94 (92.2)	6 (5.9)	2 (1.9)	0	0
Conscious sedation	6 (2.5)	28 (11.6)	77 (31.8)	81 (33.5)	50 (20.6)	17 (16.7)	64 (62.7)	20 (19.6)	1 (1.0)	0
Filling therapy tooth 16 in a 10 y.o										
How often do you use…										
Topical anesthetic	186 (77.2)	42 (17.4)	6 (2.5)	7 (2.9)	0	100 (98.0)	2 (2.0)	0	0	0
Local anesthetic	148 (61.7)	72 (30.0)	17 (7.1)	3 (1.2)	0	96 (94.1)	5 (4.9)	1 (1.0)	0	0
Conscious sedation	0	4 (1.7)	41 (17.2)	123 (51.7)	70 (29.4)	9 (8.8)	67 (65.7)	24 (23.5)	2 (2.0)	0
Extraction tooth 51 in a 4 y.o										
How often do you use…										
Topical anesthetic	224 (92.9)	9 (3.7)	4 (1.7)	2 (0.8)	2 (0.8)	101 (99.0)	1 (1.0)	0	0	0
Local anesthetic	237 (99.2)	1 (0.4)	1 (0.4)	0	0	102 (100)	0	0	0	0
Conscious sedation	75 (31.4)	76 (31.8)	40 (16.7)	20 (8.4)	28 (11.7)	66 (64.7)	34 (33.3)	2 (2.0)	0	0
Extraction tooth 14 in a 12 y.o										
How often do you use…										
Topical anesthetic	200 (82.6)	30 (12.4)	8 (3.3)	3 (1.2)	1 (0.4)	101 (99.0)	1 (1.0)	0	0	0
Local anesthetic	240 (99.6)	1 (0.4)	0	0	0	102 (100)	0	0	0	0
Conscious sedation	0	10 (4.2)	42 (17.6)	122 (51.0)	65 (27.2)	16 (15.8)	59 (58.4)	24 (23.8)	2 (2.0)	0

Responses on a Likert-type Scale; *Always*, *Often*, *Sometimes*, *Seldom*, or *Never*

**Table 3:** Comparisons between GDPs’ (General Dental Practitioners) and SPDs’ (Specialists in Paediatric Dentistry) use of pharmacological pain management and conscious sedation in four clinical scenarios

	GDPs		SPDs		*p*-value
	Always–Often	Sometimes–Seldom–Never	Always–Often	Sometimes–Seldom–Never	
	*n* (%)	*n* (%)	*n* (%)	*n* (%)	
*Scenario*					
Filling therapy tooth 55 in a 5 y.o					
How often do you use…					
Topical anesthetic	233 (95.9)	10 (4.1)	102 (100)	0	0.037^b^
Local anesthetic	220 (90.9)	22 (9.1)	100 (98.0)	2 (2.0)	0.019^b^
Conscious sedation	34 (14.0)	208 (86.0)	81 (79.4)	21 (20.6)	< 0.001^a^
Filling therapy tooth 16 in a 10 y.o					
How often do you use…					
Topical anesthetic	228 (94.6)	13 (5.4)	102 (100)	0	0.012^b^
Local anesthetic	220 (91.7)	20 (8.3)	101 (99.0)	1 (1.0)	0.006^b^
Conscious sedation	4 (1.7)	234 (98.3)	76 (74.5)	26 (25.5)	0.001^b^
Extraction tooth 51 in a 4 y.o					
How often do you use…					
Topical anesthetic	233 (96.7)	8 (3.3)	102 (100)	0	0.111^b^
Local anesthetic	238 (99.6)	1 (0.4)	102 (100)	0	1.000^b^
Conscious sedation	150 (63.0)	88 (37.0)	100 (98.0)	2 (2.0)	< 0.001^b^
Extraction tooth 14 in a 12 y.o					
How often do you use…					
Topical anesthetic	230 (95.0)	12 (5.0)	102 (100)	0	0.021^b^
Local anesthetic	241 (100)	0	102 (100)	0	
Conscious sedation	10 (4.2)	229 (95.8)	75(74.3)	26 (25.7)	< 0.001^a^

Likert-type scale replies dichotomized into *Always–Often* and *Sometimes–Seldom–Never*

^a^Chi-square test

^b^Fisher’s exact test

Summaries of direct binary logistic regressions are shown in Table 4. Regression analyses evaluated the impact of a set of predictor variables on the odds of GDPs reporting use of LA for filling therapy. Predictor variables in the model were: *sex*, *years of experience as a dentist*, *workplace*, *dentists having children of their own*, and *number of hours treating children per week*. In this model, one predictor variable (sex) had a significant impact on the odds for GDPs’ use of LA for filling therapy in a 10-year-old. However, the full model containing all predictor variables was not statistically significant (*p* = 0.147), indicating that the model was not able to make reliable predictions on use of LA among GDPs.

**Table 4:** Summary of direct binary logistic regressions

	Use of LA in a 5-year-old		Use of LA in a 10-year-old	
	OR	95% CI	OR	95% CI
*Predictor variables*				
Sex				
Male	1		1	
Female	1.686	0.643–4.423	3.030	1.118–8.210
Experience as a dentist				
≤ 5 yrs	1		1	
> 5 yrs	0.327	0.062–1.721	0.364	0.084–1.547
Workplace				
SPD	1		1	
PP	0.371	0.081–1.695	1.080	0.238–4.897
Children of their own				
No	1		1	
Yes	0.829	0.255–2.700	0.447	0.145–1.380
Time treating child patients per week				
≤ 10 h	1		1	
> 10 h	0.523	0.118–2.310	0.579	0.126–2.664

GDPs' (General Dental Practitioners) reported use of local anesthetic (LA) for filling therapy in tooth 55 in a and 5-year-old and tooth 16 in a 10-year-old (dichotomized into *Always–Often* and *Sometimes–Seldom–Never*) as dependent variables

OR = Odds Ratio, PDS = Public Dental Service, PP = Private Practitioner

## Discussion

This cross-sectional survey showed that while SPDs reported almost universal use of LA, there was an underuse among GDPs. The underuse of LA among GDPs for filling therapy in primary teeth was associated with higher mean age and more years of experience as a dentist. Furthermore, GDPs with fewer years in the profession reported spending a higher number of hours treating children per week.

The low response rate among GDPs is a concern in this study. We used the HOSP registry, held by the National Board of Health and Welfare, to identify and invite participants. This registry comprises all licensed dentists in Sweden and was chosen as being the best registry to cover the target populations. In a previous study using the same questionnaire, a different registry was used, and that study reached a higher response rate (Berlin et al. 2018). Today, that registry has a lower coverage and was therefore deemed less accurate. At the time of inclusion of GDP participants for this study, it was not possible to gain information on an individual level whether a dentist treated children or not. Since information was only available at the clinic level, all dentists working at a clinic providing child dental care were invited. It can thus be assumed that not all invited GDPs treated children and that several of these dentists are among those who have not responded.

Response rates in surveys addressed to dentists and other health professionals have declined over time (Asch et al. 1997; Cook et al. 2009) and a general fatigue to surveys has been suggested as the reason behind this (McKernan et al. 2022). This trend can be illustrated by three different digital surveys. The first two were from Sweden, one carried out in 2012 reaching a response rate of 68.3% (Krekmanova et al. 2021), the second study performed data collection in 2021–2022 and 18.6% of the invited persons responded (Fisic et al. 2024). A third study from the U.S. involved pediatric dentistry program directors and post-graduate dentists and concerned opinions about the virtual interview process for the selection of candidates in pediatric dentistry residency programs during the COVID-19 pandemic (Yeroshalmi et al. 2024). Although the topic can be regarded as important to the informants, the response rates were low, 27% and 17%, in the two groups, respectively. These examples highlight the challenges of conducting questionnaire studies and justify why the present study’s response rate of 48% must be considered acceptable.

It has been debated if the mode of distribution of questionnaires (i.e., digitally and/or by post) could affect response rates. In a systematic review, Al Khalaf et al. (2022) could not find a definite answer to this, both ways of distribution reached approximately the same response rate. For the convenience of the respondents, the present study provided the option to answer the questionnaire either by mail or digitally, which is a strength. Further, we sent out two reminders, a practice that has been described as important to bolster response rates, also a strength from a methodology point of view (Cook et al. 2009). Another strength is the questionnaire. This was based on relevant clinical scenarios familiar to all dentists and has been used previously. This enables repeated standardized cross-sectional surveys over time and in different cultural contexts on the subject. All together, we believe that the present study is in good standard and that the results are valuable for both researchers and clinicians working with children in dental care.

A notable strength of the present study is that it is based on a well-defined population. Regarding GDPs, data were collected in the county of Scania, the third most populous of Sweden’s 21 counties. The proportions of population, including children and adolescents, as well as dentists are just over 13%, and the dental health among children and adolescents in the county is similar to the national average. Furthermore, a corresponding study collected data in the same geographic area in 2014 (Berlin et al. 2018). Thus, it is likely that the study population mirror Swedish dentists, and the results are representative of the entire country. Given that the number of Swedish SPDs is limited, all clinically active SPDs were included in this study.

According to this study, SPDs almost always use LA, i.e., much more often than GDPs which is supported by previous reports from both Sweden (Berlin et al. 2018) and other countries (Schorer-Jensma and Veerkamp 2010; Lee et al. 2015). The lower use of LA in general dental practice is a dilemma. In the present study, around 90% answered that they used LA *Always/Often* for restorations. While this may appear to be a high frequency, a similar proportion of respondents selecting *Always* alone would have been a more positive outcome. Still, it is an improvement compared with Berlin et al. (2018) who reported frequencies of 77% and 86% for restorative treatment in primary and permanent teeth, respectively. Other comparable figures from the Nordic countries show a varying use of local anesthesia for restorative procedures. For instance, 88% of Danish dentists reported using LA *Always* or *Often* (Rasmussen et al. 2005). In Norway, the reported frequency ranged from 41 to 95%, depending on the child’s age (Rønneberg et al. 2015). In Sweden, Wondimu and Dahllöf (2005) found that only 30% of dentists reported never refraining from LA in children, while an earlier study by Klingberg et al. (1994) reported that 68% of all restorative treatments in children were performed with LA. Among American dentists, 32% and 45% reported *Always* administering LA before filling therapy in primary and permanent teeth, respectively (Murtomaa et al. 1996). The corresponding proportions in the present study were substantially higher, at 66.1% and 61.7%, respectively. Outside of the western hemisphere, a recent study reported that less than a fifth of a sample of GDPs from India administered LA *Always/Often* before restorative treatments in children (Kaul et al. 2021). Thus, the challenges to achieve better pain management for child patients lie within the GDPs. Further, it should be kept in mind that dental caries remains a problem also in Sweden where official statistics report that 23% of six-year-olds have a dft > 0 and 31% of 12-year-olds have a DFT > 0 (Socialstyrelsen 2024). Consequently, many children and adolescents risk undergoing cavity preparation without adequate pain management, i.e., experiencing pain with the risk of both dental behavior management problems and development of dental anxiety as consequences. From that perspective, setting a goal that all children should receive optimal pain prevention, including LA when they undergo restorative treatment is reasonable.

A related problem is the lack of official signals that LA should be ‘mandatory’ for use in child dental care. Sweden has adopted a system of National guidelines for dental care, including dental care for children (Socialstyrelsen 2022). These are based on available scientific evidence for different treatments and developed in collaboration with representatives from both academia and the dental health care professions. Unfortunately, there are no recommendations on the use of LA when treating children, which is surprising considering its central role in providing children with pain-free dental treatments. The explanation for this may be that the use of LA is taken for granted, or that the effect of LA is well-documented and therefore not included in the scope of the guidelines. Regardless of the reasons, it is most likely that the inclusion of LA in the national guidelines could have a major impact on how dental treatments are performed, and an extension to also include LA is on our wish list.

The finding of equal use of LA among GDPs when performing filling therapy in permanent and primary teeth in the present study is a promising development when compared with Berlin et al. (2018) who reported a lower use of LA in the primary dentition. The increase in use of LA in primary teeth may reflect educational and informational activities in recent years for GDPs and dental students emphasizing the importance of pain control in all child dental care, also when treating young children. The present study’s finding that older and more experienced dentists used LA less often in younger patients is similar to findings by Krekmanova et al. (2021). The reason behind this is not known, but it is troublesome if more experienced colleagues present this attitude, especially as they, based on their clinical experience, probably serve as role models for younger dentists who have graduated more recently. Tackling this probably requires a combination of both better guidelines and more continuing dental education in pediatric dentistry for GDPs who have worked longer in the profession.

In the present study, GDPs with fewer years of clinical experience more often reported that they both worked in PDS and had more working hours with child dental care compared with GDPs with longer work experience. This is in line with official statistics, showing that Swedish PDS organizations have an 86% share of child dental care and are also often the first employers of newly graduated dentists (SOU 2021; Socialstyrelsen 2023b). As less-experienced dentists have been shown to feel more stress and difficulties when treating child patients (Rønneberg et al. 2015), it is important that introductory programs for new graduates also cover pediatric dentistry and pain management. The transition from student to practicing health professional is a potential stress factor, and stress is also more frequently reported in younger dentists (Brennan et al. 2010; ADA 2022; Brennan et al. 2024). Preventing this stress is important and the process of growing into the role of being a dentist could be facilitated by providing new colleagues in GDP with the support of a mentor (Ali et al. 2016).

There is concern that undergraduate training does not prepare dental students to treat and care for younger children or children with more extensive treatment needs (Rodd et al. 2010; Walley et al. 2014; Casamassimo and Seale 2015; Casamassimo et al. 2018). This is supported by the fact that the extent of clinical training has been reduced in dental undergraduate educations in Sweden (SOU 2021). Theoretical and clinical training in pediatric dentistry, separate from orthodontics, vary in length between the four Swedish dental schools. On average, this comprises eight to nine weeks full-time education (⁓ 13 ECTS). Courses in pediatric dentistry are stretched out over the final years of education and run in parallel with other subjects. In addition, learning activities with relevance to pediatric dentistry are also found in other subjects, e.g., radiology, oral pathology, and surgery. Too little clinical training during undergraduate education could imply that newly graduated dentists are not fully prepared to treat children in general or to prevent pain in particular. Among Swedish SPDs, the dilemma of limited clinical experience among new graduates is often discussed and so is the impact of time pressure in general child dental care. It has also previously been reported that many GDPs find dental care for children to be more stressful than dental care for adults (Ayers et al. 2008; Davidovich et al. 2015; Rønneberg et al. 2015; Tokgöz Kaplan 2024).

Capitation-based reimbursement systems carry certain risks, particularly the risk that the cost of providing care may exceed the capitated revenue for patients with greater treatment needs. Inadequate compensation may, in turn, reduce providers’ willingness to deliver dental care to children and adolescents, potentially shifting a disproportionate burden of care onto organizations with responsibility of last resort. Reported effects of a capitated payment model on child dental care have included lower care output and care utilization, and a higher rate of referrals as compared to a fee-for-service model (Brocklehurst et al. 2013). In addition, time spent by dentists on each patient visit has been reported as lower in a capitated payment system (Johansson et al. 2007). This is alarming as sufficient time is crucial when treating children, especially when introducing children to dental treatments, including administering LA and to provide pain-free dental care. In recent years, the low reimbursement for child dental care has been debated within the dental profession in Sweden. This has mostly focused on the dentists’ perspective of whether it is possible to treat children in the clinic from a financial point of view. The core issue, i.e., whether this affects children's dental care, their dental health, or children’s perceptions of seeing a dentist has not been sufficiently addressed by stakeholders like politicians, the management of PDS or by specialists in pediatric dentistry. Unfortunately, these stakeholders have not taken on the roles and responsibilities of advocating for the fundamental rights of children, i.e., the right to the highest attainable standard of health (UN). It is not known how this lack of action has impacted on the work environment or stress levels among GDPs. How this affects GDPs’ pain management, and in a longer perspective also children's access to pain-free treatment, needs to be further researched.

## Conclusions

To conclude, there is still an underuse of LA in general dental practice when treating child patients in Sweden. This is troublesome as painful treatment can impact negatively on children’s perceptions of dental care, lead to avoidance, and in a longer perspective deterioration of dental health. The reasons for refraining from LA are not fully understood, but possible contributing factors can be identified within the work environment, insufficient undergraduate training and lack of organizational support and guidelines.

## Data Availability

The data that support the findings of this study are available from the corresponding author, [RR], upon reasonable request.

## Declarations

### Trial registration

The study protocol was registered in ClinicalTrials.gov (NCT05911542) on 15 June 2023.
